# Artificial Intelligence Model for a Distinction between Early-Stage Gastric Cancer Invasive Depth T1a and T1b

**DOI:** 10.7150/jca.94772

**Published:** 2024-04-08

**Authors:** Tsung-Hsing Chen, Chang-Fu Kuo, Chieh Lee, Ta-Sen Yeh, Jui Lan, Shih-Chiang Huang

**Affiliations:** 1 Department of Gastroenterology and Hepatology, Linkou Medical Center, Chang Gung Memorial Hospital, Taoyuan, Taiwan.; 2 Division of Rheumatology, Allergy, and Immunology, Chang Gung Memorial Hospital- Linkou and Chang Gung University College of Medicine, Taoyuan, Taiwan.; 3 Department of Information and Management, College of Business, National Sun Yat-sen University, Kaohsiung city, Taiwan.; 4 Department of Surgery, Chang Gung Memorial Hospital, Linkou, Taoyuan, Taiwan.; 5 Department of Anatomic Pathology, Kaohsiung Chang Gang Memorial Hospital, College of Medicine, Chang Gung University, Taoyuan, Taiwan.; 6 Department of Anatomical Pathology, Chang Gung Memorial Hospital, Chang Gung University College of Medicine, Taoyuan, Taiwan.

**Keywords:** artificial intelligence model, image classification, endoscopic ultrasound, endoscopic submucosal dissection, early gastric cancer, tumor invasion depth

## Abstract

**Background:** Endoscopic submucosal dissection (ESD) is a widely accepted treatment for patients with mucosa (T1a) disease without lymph node metastasis. However, the inconsistency of inspection quality of tumor staging under the standard tool combining endoscopic ultrasound (EUS) with computed tomography (CT) scanning makes it restrictive.

**Methods:** We conducted a study using data augmentation and artificial intelligence (AI) to address the early gastric cancer (EGC) staging problem. The proposed AI model simplifies early cancer treatment by eliminating the need for ultrasound or other staging methods. We developed an AI model utilizing data augmentation and the You-Only-Look-Once (YOLO) approach. We collected a white-light image dataset of 351 stage T1a and 542 T1b images to build, test, and validate the model. An external white-light images dataset that consists of 47 T1a and 9 T1b images was then collected to validate our AI model. The result of the external dataset validation indicated that our model also applies to other peer health institutes.

**Results:** The results of *k*-fold cross-validation using the original dataset demonstrated that the proposed model had a sensitivity of 85.08% and an average specificity of 87.17%. Additionally, the *k*-fold cross-validation model had an average accuracy rate of 86.18%; the external data set demonstrated similar validation results with a sensitivity of 82.98%, a specificity of 77.78%, and an overall accuracy of 82.14%.

**Conclusions:** Our findings suggest that the AI model can effectively replace EUS and CT in early GC staging, with an average validation accuracy rate of 86.18% for the original dataset from Linkou Cheng Gun Memorial Hospital and 82.14% for the external validation dataset from Kaohsiung Cheng Gun Memorial Hospital. Moreover, our AI model's accuracy rate outperformed the average EUS and CT rates in previous literature (around 70%).

## Introduction

Gastric cancer is a significant health concern both in Taiwan and worldwide 1-8. Therefore, understanding and addressing gastric cancer incidence and mortality rates is paramount. Such knowledge can aid in the early detection and treatment of these cancers, improving survival rates, reducing healthcare resource wastage, and facilitating the development of more effective prevention strategies. Endoscopic Submucosal Dissection (ESD) is a valuable treatment procedure for excising lesions once Early Gastric Cancer (EGC) or high-grade dysplasia is confirmed. ESD allows for lesion removal while preserving the organ and promoting a good quality of life for the patient. However, accurate staging of EGC is crucial for determining the appropriate treatment approach. Conventionally, Endoscopic Ultrasound (EUS) and Computed Tomography (CT) scans have been widely used for gastric cancer (GC) staging. Unfortunately, these tools must be scheduled after the first endoscopy examination, increasing the effort and financial burden on the healthcare institution and the patient. This study aims to implement an artificial intelligence (AI) method to build a model for classifying the invasive depth of early GC without using ultrasound and/or CT scans. With such an AI model, physicians can detect and remove early gastric cancer in a single endoscopy treatment.

We studied EGC in the stages of mucosa (T1a) and submucosa (T1b). These are both early cancers in which no evidence of lymph node metastasis appears. Unlike T1b, T1a can be removed by endoscopic resection, also known as endoscopic submucosal dissection (ESD) treatment, in which the endoscopist first locates the lesion and then uses stage classification methods to classify the invasion stage after the endoscopy section. The T1a lesion is then removed in the next endoscopy section. Thus, if they could first determine whether the cancer is T1a or T1b, the physician could locate and treat T1a during a single endoscopy without using other classification methods. Although T1a and T1b are challenging to distinguish, early cancer detection can increase the survival rate significantly; patients with EGC have a five-year survival rate of more than 95% after receiving appropriate therapy, resulting in an improved quality of life 2.

Various staging methods are used for image classification following the detection of a potential lesion by endoscopy, including CT scans, EUS, and positron emission tomography 1. However, these methods are all applied following endoscopy treatment, and removing a T1a lesion requires another section of endoscopy after staging. Furthermore, CT has a low sensitivity rate, and EUS has a low accuracy of approximately 70% for EGC staging 4. The accuracy range of EUS varies significantly owing to inconsistent operating physician training quality, making it necessary to develop a stable and objective investigative image classification method. Although AI models have been applied in numerous medical fields, their use in tumor-depth studies has been limited. Our study was one of the few intending to develop an AI tool for tumor-depth classification in EGC.

AI models have been successfully applied in numerous medical fields, revolutionizing diagnostics, treatment planning, and prognosis prediction. In an approach similar to that of this study, Tokai et al. 9. developed an AI model for tumor depth classification in esophageal squamous cell carcinoma. They also attempted to remove early cancers during endoscopy treatment and to find an AI model that could effectively assist endoscopists. Jing et al. 10 conducted an extensive review of AI applications to GC and concluded that the accuracy of such AI models was promising. Their review found limited use of AI for tumor depth assessment in T1a and T1b 9. Kubota et al. 11 proposed a model to classify GC invasion depth, but its accuracy for T1a and T1b discrimination was low (68.9 and 63.9% for T1a and T1b, respectively). The present study explored how to utilize AI and data augmentation to improve the accuracy of tumor depth assessment in T1a and T1b. Using a white-light image database compiled from a hospital's cases of early GC (351 and 542 images for T1a and T1b, respectively), an AI model was constructed to automatically analyze and automatically evaluate the tumor depth and staging of T1a and T1b.

The results of this research provide the clinical community with a rapid, reliable, and non-invasive method to assist physicians in accurately assessing tumor invasion depth and treating EGC patients in a single endoscopy section. This will benefit patients by avoiding unnecessary surgeries and treatments while improving the success rate of ESD.

## Methodology

### Data Collection

**Figure 1:**
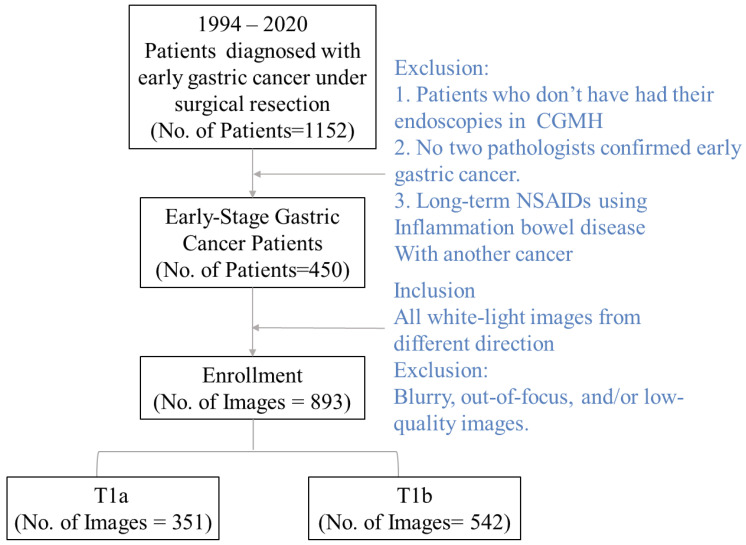
The individual's endoscopy white light image exclusion and inclusion flow chart.

We collected a dataset of white-light images consisting of 351 T1a and 542 T1b images from one of the leading research hospitals in Taiwan, Cheng Gung Memorial Hospital, Linkou. We first collected all images of those who underwent gastroscopic biopsies in the hospital between 1994 and 2020. Since the images are collected exhaustively, the collection process is not randomized. After all the images were collected, we excluded them according to the following criteria (see Fig. 1). First, the patients who did not complete their endoscopy in the FGMH were excluded from the dataset. Second, the patient's lesion must be staged. Diagnostics by two pathologists and the image staging had to be early-stage gastric cancer. Note that physicians might take more than one image per lesion. All images from lesions not staged as early cancer are excluded from the data set.

Furthermore, we excluded all blurry or out-of-focus images, and photos with poor image quality were deleted. The data comprised white-light images collected solely from the Cheng Gung Memorial Hospital (CGMH) database (IRB number 20210151B0). Note that our model-building images are exclusively collected from the individual's visit to Cheng Gung Memorial Hospital, Linkou. In addition to the model-building dataset, we collected an external validation dataset from Kaohsiung, Cheng Gung Memorial Hospital, with the same inclusion and exclusion procedure. Our validation dataset consists of 47 T1a and 9 T1b white-light images. While all white-light images go to the same database in the Cheng Gung Memorial Hospital, those two branches service patients in different geographic regions and act as individual profit centers.

The descriptive statistics of the included individual's demographic information and their medical history. Table 1 summarizes the individual's age, sex, and whether the individual suffers from other gastric ulcer disease. We also report the location of the lesion and resection margin, along with the pathological result of ultrasound stage (N), stage, and histology results. In the univariate analysis of the available data, we find the difference in the percentage of the T1a and significant concerning the location of the lesion, gastrectomy, carcinoembryonic antigen (CEA), peptic ulcer infection conditions, or resection margin. This indicates that the invasion depth is not significantly related to those factors.

**Figure 2:**
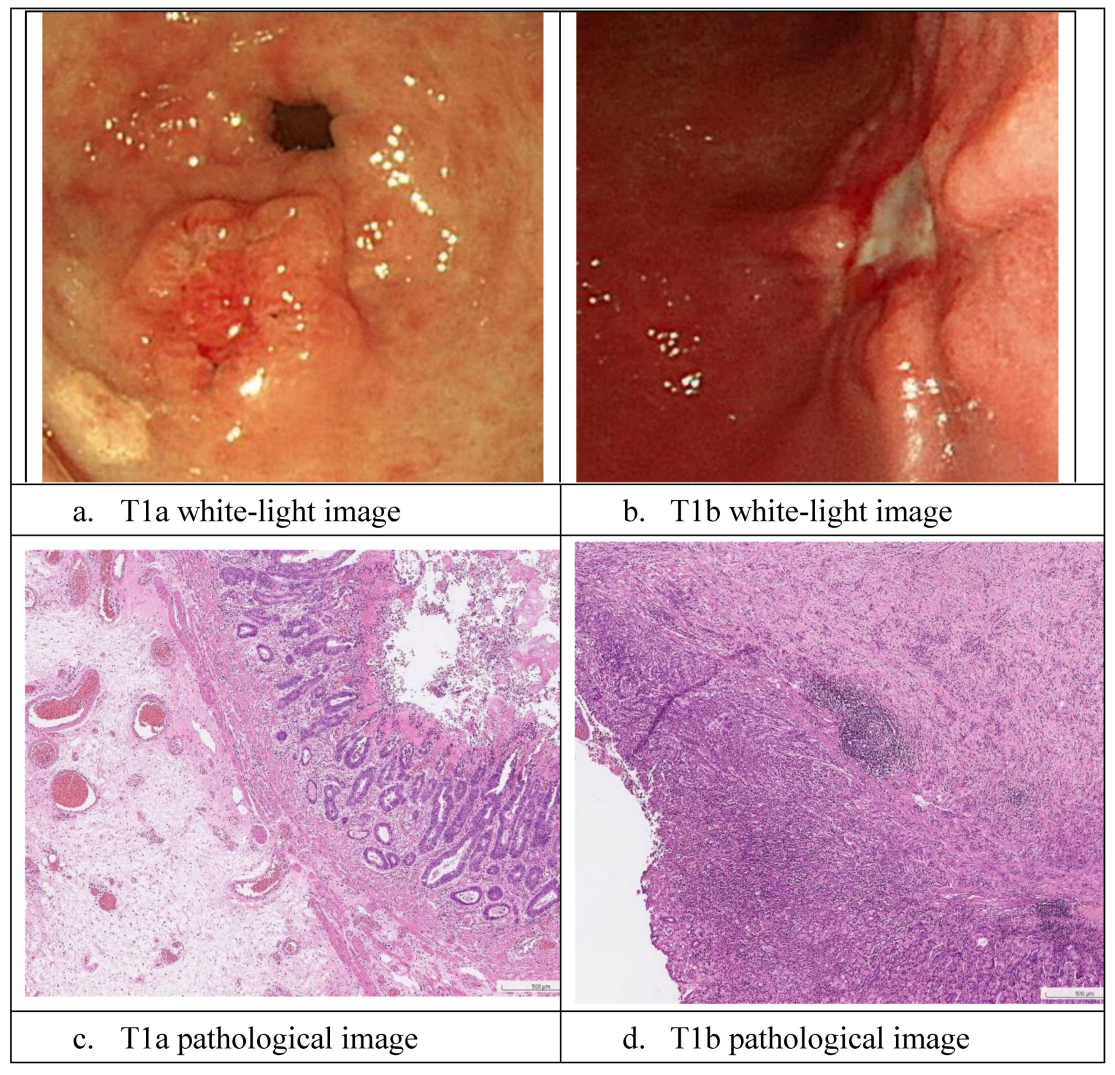
White-light and pathological images of gastric early cancer.

Sample white-light images of T1a and T1b are shown in Fig. 2. As it is difficult to classify these cancers during the endoscopy process, the EUS and CT scans were not 100% reliable, and the true state of T1a and T1b classification in our dataset was based on pathology reports. In this section, we describe the development of the proposed AI model. Moreover, in the following sections, we present our results and statistical analysis. The pathological images of the images above are provided in the following Fig. 2 c and d. The pathological images of the images above are provided. As shown in the graph in Fig. 2c, T1a-stage cancer cells only invade the mucosal layer, and the Paris endoscopic classification is type IIa. In Fig. 2d, the T1b cancer cells invade into the submucosa layer without involving the muscularis propria and The Paris endoscopic classification is IIa+c.

**Figure 3:**
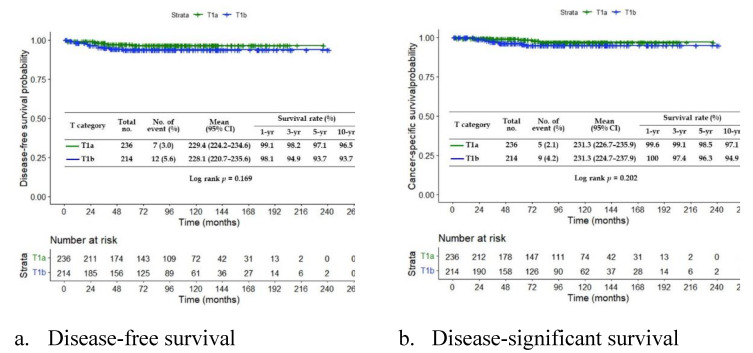
The survival analysis of treated EGC patients

We also calculate the survival rate for the T1a and T1b patients. Our survival analyses show that the early treatment of EGC can significantly increase the patient's survival rate. We present the disease-free survival (Fig. 3a) and disease-significant survival (Fig. 3b). Both analyses show the constant decay of the number of patients at risk. At month 264, all patients are diagnostic as risk-free.

### Data Augmentation and YOLO V4

To enhance the classification model using our dataset, we propose a hybrid approach involving data preprocessing and image recognition. We implemented the data augmentation *Algorithm 1* to improve the feature extraction process and address the issue of limited sample size.

**Figure 4:**
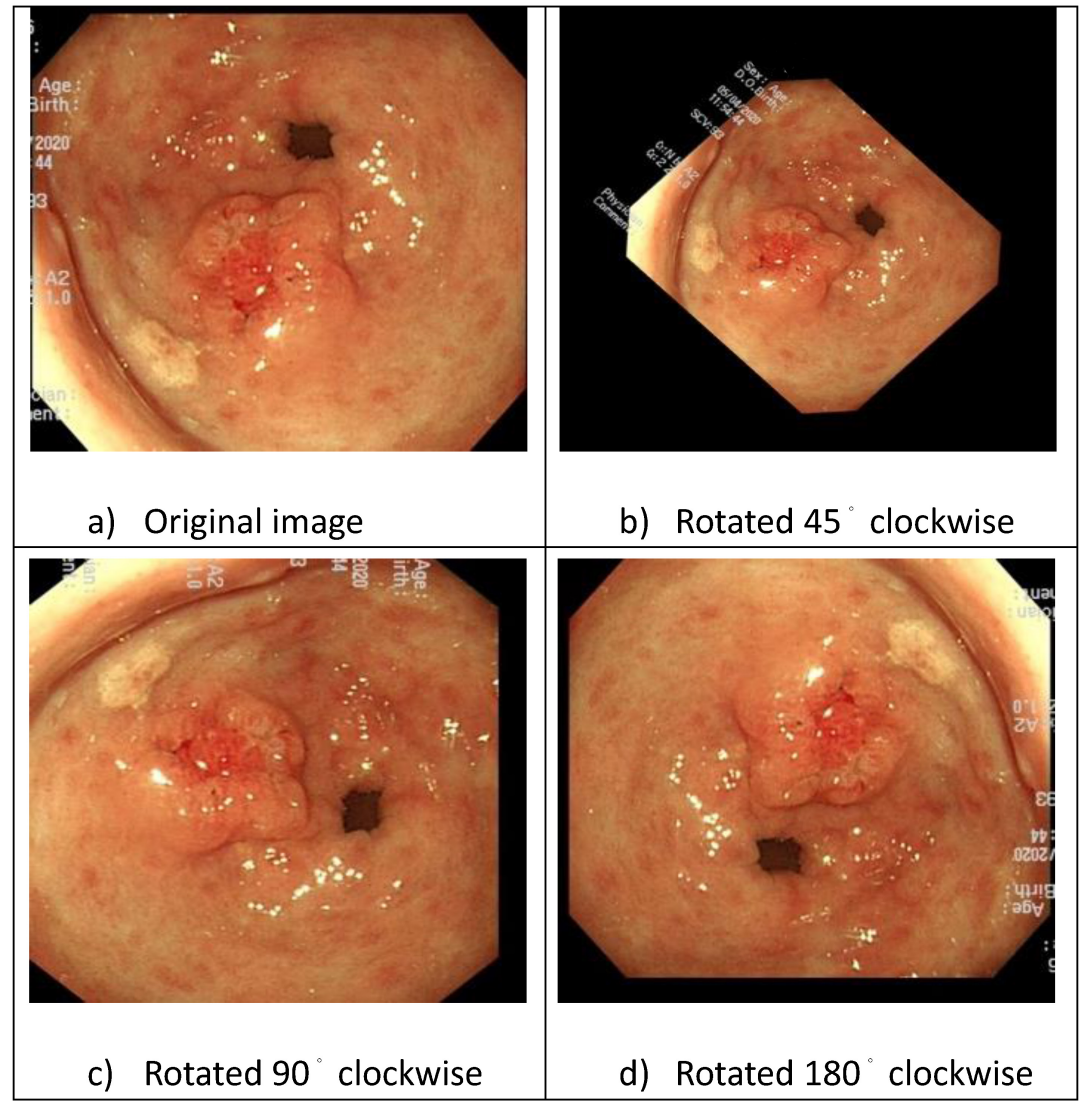
Image rotation and enhancement.

First, we classified the images into T1a and T1b based on pathological reports. Subsequently, the physician marked the lesion in each image using “You Only Look Once" (YOLO). The images were augmented by applying rotations using XnConvert (Fig. 4), and brightness adjustments were made using image enhancement software to improve the efficiency of feature extraction.

Our AI model, Heuristic, is a two-step method. In the first step, four-fold validation is implemented to determine the model's batch size, subdivision, and learning rate. The four-fold method is efficient and does not require extensive running time. The four-fold method approach used by Heuristic is summarized in ***Algorithm 1***.

Index

*i* = subscript of images, from 1 to n,

*j* = level of brightness, from 1 to 3,

*z* = degree of rotation, from 1 to 4,

*k* = index of the combination used for model building data sets, from 1 to *k*.

**Algorithm 1: *Four-fold method*:**

Step 1: Construct **deep-learning datasets** based on the four-fold method. The original dataset is randomly divided into four equally sized subsets labeled sets 1, 2, 3, and 4. For each run, two subsets are used as the modeling dataset and one as the testing dataset. Each run is represented using labels of the modeling, testing, and validation datasets. For example, a run with datasets 1 and 2 as the modeling dataset, 3 as the test dataset, and 4 as the validation dataset is named 1234 and indexed with the number *k*. The remaining data are used as validation datasets.

Step 2: For the modeling and testing data sets, process* image_i_* by adjusting the brightness into three levels original (*j=*1), dark(*j=*2), and bright(*j=*3). Output data set [*image_ij_*] of size nX3.

Step 3: Augment the set [image_ij_]by rotation by degree original (*z=*1), 45° (*z=*2), 90° (*z=*3), and 180° (*z=*4), and output the augmented data set [*image_ijz_*]

Step 4: Label the actual state of [*image_ijz_*] with YOLO V4 and output the labeled data set.

Step 5: Input all datasets into *Subroutine A*.

Step 6. Collect output from *Subroutine A* and calculate the discrimination indices.

***Subroutine A***:

Step 1: Initiate *k*=1 and go to Step 2.

Step 2: Input dataset *k* into YOLO V4. Go to Step 3.

Step 3: Find the best parameters (learning rate, batch size, and subdivisions) for the AI model and output the resulting model.

Step 4: Output the results of the testing and validation data set from YOLO V4, store it as results of model*_k_*.

Step 8: Collect the results. If *k*= 12, break from *Subroutine A* and output all testing results of [model*_k_*]. Otherwise, set *k* = *k* + 1 and return to Step 2.

After the batch size, subdivision, and learning rate are fixed, *k*-fold validation is used to test the effectiveness of the model. *Algorithm 2* summarizes this *k*-fold method.

**Algorithm 2: *k-fold method***

Step 1: Initialize by setting *k*=1.

Step 2: Process all images except *image_k,_* keeping it as the testing image. Process* image_i_* by adjusting the brightness into three levels: original (*j=*1), dark(*j=*2), and bright(*j=*3). Output data set [*image_ij_*] with size nX3.

Step 3: Augment the set [*image_ij_*]by rotation by degree original(*z=*1), 45° (*z=*2), 90° (*z=*3), and 180°(*z=*4) and output the augmented data set [*image_ijz_*]

Step 4: Label the actual state of the [*image_ijz_*] with YOLO V4 and output the labeled data set.

Step 5: Input [*image_ijz_*] as modeling set and build an AI model with parameters of the best model, i.e., the model with the highest accuracy under the four-fold method, and then go to Step 6.

Step 6: Input *image_k_* as the testing image, output the testing result, and then go to Step 7.

Step 7. Record the output results in dataset** [testing]** and set *k = k*+1. If* k*+1 =804 go to Step 8; otherwise go to Step 2.

Step 8. Collect output from **[testing]** and end the algorithm.

YOLO combines the location of the lesion and the classification of the selected image. In particular, YOLO is an AI model that relies on convolutional neural networks (CNN). It differs from other AI models, such as TensorFlow, as it automatically marks the location of defects. In this case, the YOLO model automatically located a lesion. The YOLO model comprises three parts: backbone, neck, and head. The backbone uses CSPDarknet 53, which has 53 CNN layers, to extract features from the images. The features were then reconfigured into feature maps by the neck. Finally, the head uses these to locate and classify the images.

### Statistical methods

We implemented a four-fold cross-validation to validate and search for the optimal parameters of the AI model. To further validate the proposed method and avoid overfitting, we used the k-fold method to validate the model with optimal parameters given by the four-fold method. The four-fold cross-validation was used to illustrate the consistency of the proposed method. For each dataset, we calculated the overall accuracy of the validation dataset and the 95% confidence interval (CI) for accuracy. To assess the applicability of our model, we used the same AI model to stage a lesion image obtained from an external healthcare institution using four-fold cross-validation.

In this study, we classified T1a as 1 (positive) and T1b as 0 (negative). A confusion matrix was constructed, as shown in Table 2. When our AI model classifies image A as T1a, we recorded the predicted state of image A as “positive.” If the pathological record of image ***A*** is also T1a, then the image ***A*** is recorded as “true positive”. This indicated that our predicted “positive” state and pathological result are identical. In contrast, if the pathological record of image A is T1b, then image ***A***'s prediction is “false positive.” The “false positive” indicates that the AI model's prediction is inconsistent with the pathological record. The same logic can be applied when the AI model classifies the image as T1b (negative). The performance of the AI model is demonstrated by sensitivity (1), specificity (2), and accuracy rate (3), are presented in the results section. In our case, the sensitivity indicates the efficiency of the AI model correctly identifying the T1a. In complement, the specificity suggests the efficiency of determining the T1b. We also included a 95% CI to illustrate the model consistency. The external model valuation was designed to use the model built by randomly selecting 75% among all white-light images and the remaining 25% as a testing data set. The external dataset was then used as the validation dataset.


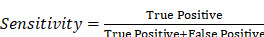

(1)


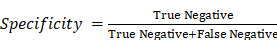

(2)


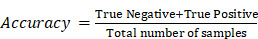

(3)

Where total sample size equals 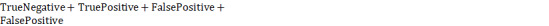.

**Figure 5:**
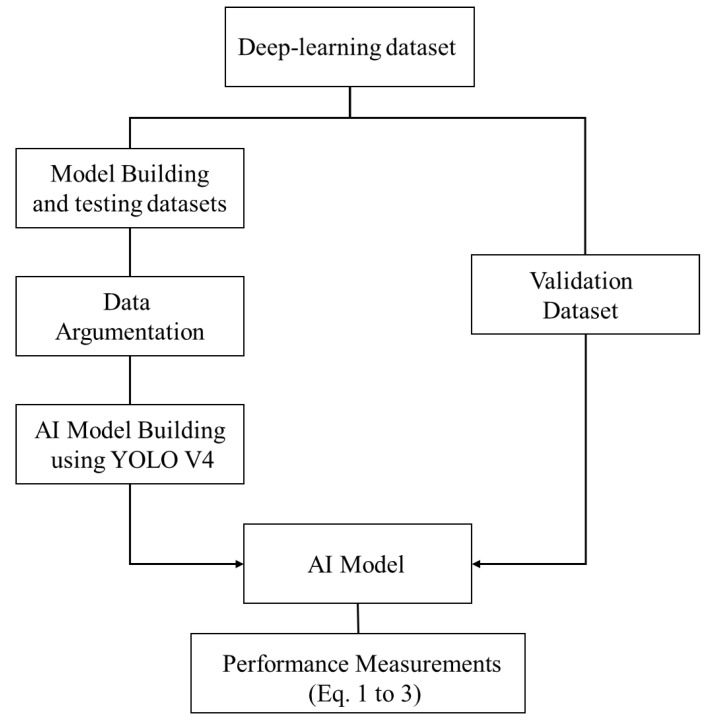
The general AI model validation procedure.

The model building and validation procedure are presented in Fig. 5. The sensitivity, specificity, and accuracy are calculated based on equation 1 to 3. Note that the validation data set does not go through the data argumentation process.

## Results

### Experimental Results and Validation of Original Dataset

We adopted YOLOV4. We could obtain the best classifying model with a batch size of 64, a learning rate of 0.0001, and a maximum batch size of 4000. Our AI model outputs the predicted state for each image in the validation dataset as described in Algorithm 1 Step 4. The result is then compared with a pathological record for each image and output confusion matrix as described in Table 1 and Fig. 5. We calculated the AI model performance measurements such as sensitivity, specificity, and accuracy as described in equations 1 to 3. The validation results of our 12-subdivision datasets are summarized in Table 3. The overall average accuracy rate was 76.88%, and the 95% CI ranged from 75.17% to 78.60%, better than the EUS accuracy rate. The average sensitivity was 80.09%, and the 95% CI ranged from 78.91 to 82.90%. The average specificity was 77.04%, and the 95% CI ranged from 75.76% to 78.31%.

To complement the white-light images, we also built the AI model using narrow-band imaging (NBI) images; however, the NBI results did not outperform the white-light results, indicating that our model did not require the supplementary use of NBI to improve its classification accuracy. It is worth noting that, even at its lowest accuracy, our AI model outperformed models in the previous literature in terms of accuracy by 60 to 70% 4, 11. Furthermore, using YOLO V4, our AI model could classify the invasion stage and locate tumors for the endoscopist using only white-light images. Those functionalities significantly reduced the time and effort needed for ESD.

Once the parameters were obtained using the four-fold method, we conducted a **k-fold test** to test whether the model suffered from overfitting. The results of the k-fold study are summarized in Table 4. In k-fold validation, overall accuracy, sensitivity, and specificity are all higher than the four-fold results, indicating that our AI classification approach is a consistent and reliable model for classifying early cancers such as T1a and T1b. Furthermore, with a small sample size, we reached an accuracy of almost 90%, another significant contribution of our approach to building an AI classification model.

### Validation Results from External Data Set

Although our AI model exhibited promising results, it might be prone to overfitting when images from a single hospital are used. To address this issue, we collected validation data consisting of 47 T1a and 9 T1b images from Kaohsiung Cheng Gun Memorial Hospital. We used the AI model built using 75% of our original data set and tested it with the remaining 25% of the data. Images from Kaohsiung Cheng Gun were used as the validation dataset. The results are shown in Figure 3. In the validation results, sensitivity is 82.98%, specificity is 77.78%, and overall accuracy is 82.14%. The result outperformed the four-fold case obtained using the original dataset as the validation dataset. This indicates that our AI model is not overfitted and is suitable for hospitals other than Linkou Chang Gun Memorial Hospital. The result produced by the external dataset demonstrates that our method can be applied to hospitals worldwide and that our results are reproducible. Hence, any peer healthcare institution can implement the proposed AI model build an algorithm and build their AI classification model enterally in the institution.

## Discussion

ESD can be performed to excise the lesion once EGC or high-grade dysplasia is confirmed while preserving the organ and promoting a good quality of life 12, 13. EUS and CT scans are widely used for GC staging. However, these two tools exhibit low accuracy in staging EGC, ranging from 45% to 92% 3,9-13. Additionally, a CT scan is insufficient to assess the invasive tumor depth between the mucosa and submucosa 14. This is especially problematic because the depth of invasion in the mucosa and submucosa layers is critical for treatment decisions, and an ECG and/or CT scan might not provide sufficient information in this regard.

Several factors contribute to the challenges in accurately staging EGC with EUS. The factors affecting the inspection accuracy of EUS include the presence of ulcers in lesions 15-17, the location of the lesion in the upper third of the stomach, the presence of fibrotic tissue, and larger tumor size 18. Additionally, the experience and skill of the endoscopist play a significant role in the accuracy of staging EGC. Therefore, an objective, unbiased diagnostic tool is required. AI models have emerged as a promising solution to addressing these challenges and the need for an objective and unbiased lesion staging tool. AI classification models have widespread applications in various domains, including the medical field. AI offers the advantage of providing a stable and consistent diagnostic quality that is not influenced by subjective factors. AI classification of T1a and T1b can deliver rapid and cost-effective results during the endoscopy section, which can help mitigate the costs associated with additional EUS and second endoscopy sections. Thus, implementing an AI model in staging T1a and T1b can reduce the need for endoscopy, EUS, and CT scans, thereby reducing the burden on healthcare institutions and patients.

Although AI is being increasingly applied in various healthcare problems, the data needed for AI model building remains a top issue. From an AI modeling perspective, we combined data augmentation with YOLOV4. We constructed an effective AI model with a relatively small sample size by applying simple data augmentation and image enhancement techniques. Unlike abandoned images from repetitive production processes in the healthcare industry, many early cancer and precancerous lesion databases are relatively small. The proposed method, which combines simple data argumentation and AI modeling, can provide a new approach for future researchers in AI modeling with small datasets. In this respect, our research is particularly relevant for situations where early cancer and precancerous lesion databases are limited. Our study contributes to developing AI modeling for rare diseases and conditions with constrained image access due to data source limitations.

In summary, ESD is a valuable method for excising lesions while preserving organs and improving quality of life. However, accurate staging is crucial for treatment decisions, and conventional methods such as EUS and CT scans have limitations. AI provides a promising solution by offering objective and consistent diagnostic capabilities, ultimately contributing to more effective and cost-efficient healthcare. As aforementioned, the endoscopist can classify T1a and T1b during the endoscopy section with our AI model. Therefore, one doesn't need an additional EUS or CT for the treatment. In Taiwan, the National Health Insurance sets the cost of EUS at 5029 New Taiwanese Dollar/section (about $167/section) and takes 10 to 15 minutes. The cost and time of diagnostic T1a and T1b using the AI model are trivial once the model is embedded in the system. Furthermore, the cost of EUS in EUS in other countries, such as the United States, ranges from $1250 to $4800/section 19. Hence, if the AI model can be applied to those higher-cost countries, our AI model will significantly reduce the cost and risk of diagnosing and removing ECGs such as T1a and T1b.

The proposed AI model enables the classification of T1a and T1b quickly and accurately, improving patient treatment outcomes and reducing medical costs. Furthermore, AI is not influenced by subjective factors and can provide stable diagnostic quality in various situations. This will help address rising healthcare costs, making healthcare more affordable and efficient.

## Conclusion

EGC treatment is critical in increasing patient life expectancy and improving quality of life. However, accurately staging EGC between T1a, which ESD can remove, and T1b, which ESD cannot remove, is especially important. To our knowledge, there is currently no efficient method for accurately classifying T1a and T1b using white-light images alone. Our results show that the AI model could provide a stable, accurate, fast, and inexpensive method for diagnosing EGC. The average accuracy and 95% CI for the T1a and T1b classifications were similar to the average accuracy of current methods, such as EUS. However, our AI model can be applied to real-time endoscopic diagnosis, enabling endoscopists to decide whether a lesion should be removed. Using an external dataset, we demonstrated that the proposed method creates a consistent model that can be applied to different healthcare institutes, increasing the proposed model's applicability to peer healthcare institutions.

From a healthcare institute practitioner's perspective, our findings suggest that the AI model could accurately classify T1a and T1b lesions during endoscopic treatment, replacing conventional staging methods. Using our AI model, treating EGC with endoscopy can be more time- and cost-efficient, allowing patients/healthcare institutes to save time and cost in carrying out EUS. Additionally, the AI model frees up EUS and/or CT scan capacity for more patients in need, improving the efficiency of the healthcare institute and increasing patient welfare.

From a healthcare researcher's perspective, our AI model demonstrates an approach to address the issue of how to build an efficient AI model to diagnose rare diseases with small data sets. In addition, using white-light images alone increases the model's applicability by avoiding the need for enhanced imaging approaches such as linked color imaging (LCI). In summary, our AI model contributes to both practice and further research development.

While this study provides an efficient AI model, it has limitations. First, this study relies on images; future researchers might consider building an AI model for a real-time endoscopy section. Furthermore, researchers might consider further enhancing the accuracy by developing a new deep-learning algorithm or improving existing deep-learning methods. For example, one can modify a polyp classification model such as Polyp-PVT 20, to classify early cancer.

## Figures and Tables

**Figure 6:**
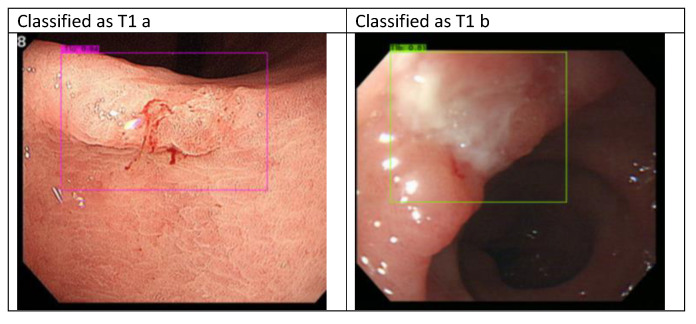
The classification results for the external data set.

**Table 1:** Demographics of patients with T1 gastric cancers following gastrectomy

Variables	No.	T1a (n=236)	T1b (n=214)	P value
Age (years)				0.921
≤65	226	118 (50.0%)	108 (50.5%)	
>65	224	118 (50.0%)	106 (49.5%)	
Sex				0.218
Male	270	148 (62.7%)	122 (57.0%)	
Female	180	88 (37.3%)	92 (43.0%)	
Peptic ulcer				0.770
No	333	176 (74.6%)	157 (73.4%)	
Yes	117	60 (25.4%)	57 (26.6%)	
CEA (ng/mL)*				0.672
≤5	382	200 (91.7%)	182 (92.9%)	
>5	32	18 (8.3%)	14 (7.1%)	
Gastrectomy				0.231
Total	55	33 (14.0%)	22 (10.3%)	
Subtotal	395	203 (86.0%)	192 (89.7%)	
Location				0.409
Upper	54	32 (13.6%)	22 (10.3%)	
Middle	115	63 (26.7%)	52 (24.3%)	
Lower	274	138 (58.5%)	136 (63.6%)	
Anastomosis site	7	3 (1.2%)	4 (1.8%)	
Resection margin				0.125
Negative	446	232 (98.3%)	214 (100.0%)	
Positive	4	4 (1.7%)	0	
N category				<0.0001
N0	372	216 (91.5%)	156 (72.9%)	
N1	50	13 (5.5%)	37 (17.3%)	
N2	19	4 (1.7%)	15 (7.0%)	
N3a	6	1 (0.4%)	5 92.3%)	
N3b	3	2 (0.8%)	1 (0.5%)	
Stage				
I	422	229 (97.1%)	193 (90.2%)	0.003
II	25	5 (2.1%)	20 (9.3%)	
III	3	2 (0.8%)	1 (0.5%)	
III				
Histology				0.026
Differentiated	235	135 (57.2%)	100 (46.7%)	
Undifferentiated	215	101 (42.8%)	114 (53.3%)	
Lymph vascular invasion				<0.0001
No	406	232 (98.3%)	174 (81.3%)	
Yes	44	4 (1.7%)	40 (18.7%)	
Perineural invasion				0.024
No	445	236 (100%)	209 (97.7%)	
Yes	5	0	5 (2.3%)	

* Not all data are available.

**Table 2:** Illustration of the confusion matrix

Confusion Matrix		Predict	
Actual		T1a	T1b
	T1a	True Positive	False Negative
	T1b	False Positive	True Negative

**Table 3:** Validation results

Model	T1a accuracy	T1b accuracy	Average accuracy	Sensitive	Specificity
2314	75.34%	82.55%	78.95%	84.85%	75.50%
2341	71.91%	77.13%	74.52%	74.55%	77.02%
3421	79.21%	83.19%	81.20%	84.30%	80.58%
3412	71.33%	79.53%	75.43%	80.70%	77.35%
1234	76.54%	79.63%	78.08%	79.44%	76.83%
1243	75.61%	85.89%	80.75%	87.61%	79.41%
1324	77.82%	83.81%	80.81%	82.24%	79.24%
1342	71.37%	76.77%	74.07%	80.70%	77.35%
1423	73.57%	79.77%	76.67%	79.74%	75.18%
1432	70.86%	75.01%	72.93%	77.02%	74.82%
2413	74.27%	78.85%	76.05%	79.01%	78.58%
2431	68.27%	78.02%	73.14%	80.70%	72.62%
Avg.	73.84%	80.01%	76.88%	80.91%	77.04%
Std	3.21%	3.23%	3.03%	3.52%	2.25%
95%CI upper	75.66%	81.84%	78.59%	82.90%	78.31%
95%CI lower	72.02%	78.18%	75.17%	78.91%	75.76%

**Table 4:** Validation results

		Average accuracy	Sensitive	Specificity
**k-fold Method Results**	Avg.	86.18%	85.08%	87.17%
	95%CI upper	88.56%	87.54%.	89.49%
	95%CI lower	83.79%	82.61%	84.86%
